# Giant Sacrococcygeal Teratoma in a Neonate: A Case Report

**DOI:** 10.31729/jnma.8251

**Published:** 2023-08-31

**Authors:** Anup Bista, Suson Ghimire, Niharika Sharma Gaire, Pujan Bataju, Dipesh Mishra

**Affiliations:** 1 Department of Anaesthesia and Critical Care, Patan Academy of Health Sciences, Lagankhel, Lalitpur, Nepal; 2 Department of Intensive Care Unit and Neurosurgery, Metrocity Hospital, Srijana Cnowk, Pokhara, Nepal; 3 Department of Intensive Care Unit, Chirayu National Hospital and Medical Institute, Basundhara, Kathmandu, Nepal

**Keywords:** *anaesthesia*, *case reports*, *neonate*, *teratoma*

## Abstract

Sacrococcygeal teratomas are common tumours in neonates and infants, primarily affecting females. A 35-year-old primigravida presented with a large sacrococcygeal teratoma that was detected during the 30^th^ week of gestation in the fetus. The baby was delivered via elective caesarean section at 36+3 weeks, and surgical excision of the 10x10x5 cm^3^ mass was performed successfully on the third day of life. Despite a surgical site infection, the patient had a favourable outcome with normal vital signs, bowel, bladder, and lower extremity functions upon discharge. Early diagnosis and prompt management of sacrococcygeal teratoma in newborns is vital for optimal outcomes, providing valuable insights and guidance to medical practitioners.

## INTRODUCTION

Sacrococcygeal teratoma (SCT), a type of extragonadal germ-cell tumour in neonates and infants with an incidence of 1 in 40,000 births, mainly affects females (3:1 ratio).^1^ These tumours arise from germ cells and are predominantly found at the base of the coccyx.

Most sacrococcygeal tumours are benign and cystic, while 1-2% exhibit malignancy.^2^ Their high vascularity can complicate pregnancy and postoperative care.

Prenatal and perinatal complications are common, requiring optimal obstetric and surgical management.^3^ This case report showcases the successful resection of the tumour which is important to prevent complications and ensure a favourable prognosis of a neonate.

## CASE REPORT

**Figure 1:**
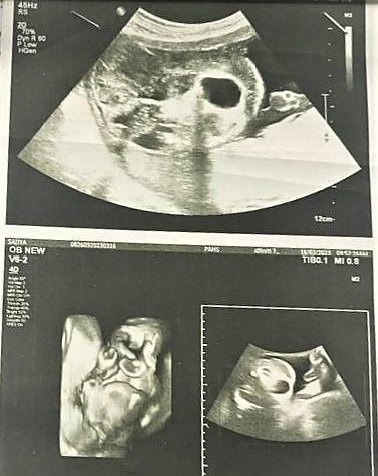

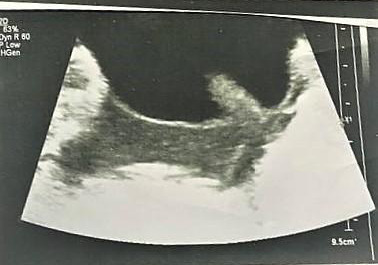
An in-utero ultrasonography at 30 weeks of gestation showing multiple cystic areas.

A 35-year-old primigravida, who had been regularly visiting our centre for antenatal care, underwent initial dating scans and anomaly scans that showed normal results. At the 30th week of gestation, an ultrasound revealed a large, heterogeneous mass lesion measuring approximately 10.3x7.4x9.7 cm^3^. It exhibited multiple cystic areas with internal vascularity which was an incidental finding (Figure 1).

The lesion originated from the caudal aspect of the fetus in the sacral region. It was exophytic and appeared separate from the abdomen and umbilical cord, suggesting a sacrococcygeal mass (teratoma). At 31 weeks of gestation, fetal magnetic resonance imaging (MRI) revealed a single intrauterine pregnancy with a large, complex cystic lesion exhibiting an exophytic component in the sacrococcygeal region. A small presacral component was also present, consistent with a sacrococcygeal teratoma (type I). There was no significant family history of congenital birth defects or genetic disorders.

**Figure 2:**
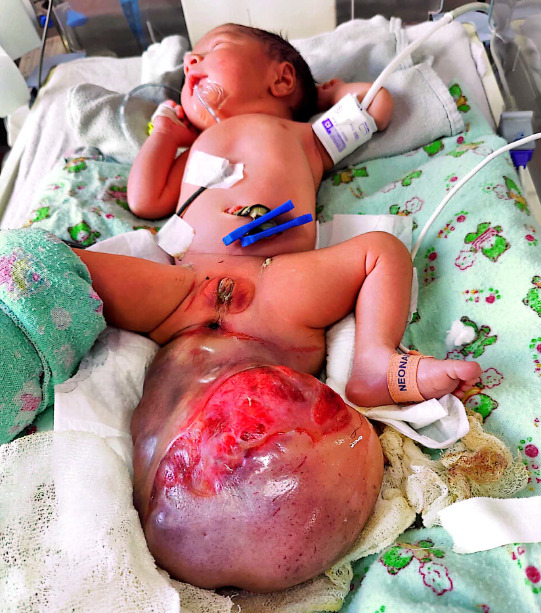
Neonatal female with huge sacrococcygeal teratoma.

A follow-up ultrasound performed at 34 weeks gestation, revealed an increase in the size of the mass. After a perinatal consultation at 36+3 weeks of gestation, the baby was delivered via elective caesarean section. The birth weight of the baby was 3370 gm, and the APGAR scores were normal. Immediately after birth, the baby was transferred to the neonatal intensive care unit (NICU) for observation (Figure 2).

**Figure 3:**
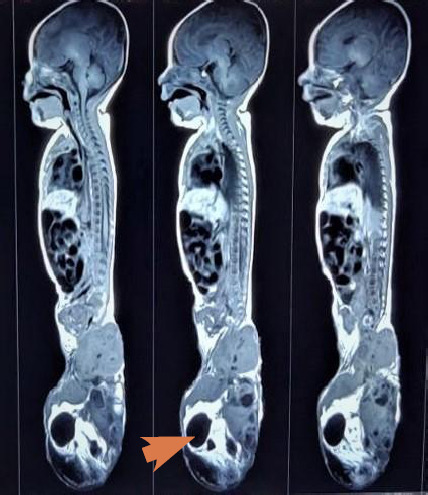
MRI of the whole neuraxis (brain and spine) showing multiloculated solid cystic mass at the sacral region.

After the baby's birth, a neurosurgical consultation was conducted to plan the excision of the sacrococcygeal mass and cover the defect. A pre-operative pediatric echocardiography revealed a patent foramen ovale with mild pulmonary artery hypertension. Abdominal ultrasonography (USG) showed normal results. A whole neuraxis MRI was performed, which revealed a multiloculated solid cystic mass arising from the lower vertebral end involving sacrococcygeal region with predominantly extra fetal portion, the small presacral component containing multiple cysts, and fat component without restricted diffusion (Figure 3).

**Figure 4:**
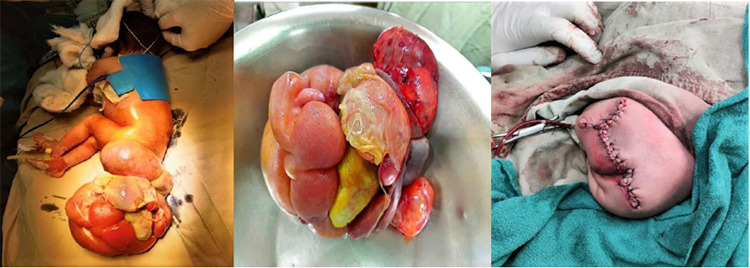
A) Intraoperative finding with sacrococcygeal mass, B) Excised sacrococcygeal gross mass, C) After surgical reconstruction on the 3rd day of life.

On the third day, excision of the sacrococcygeal mass was performed under general anaesthesia in a prone position which lasted for approximately four hours. Caudal analgesia was not administered due to the presence of the mass. Pain management was done with paracetamol and fentanyl. Additionally, neonatal considerations such as fluid management and temperature were closely monitored. The total blood loss amounted to approximately 50 to 70 ml, and 40 ml of blood was transfused intraoperatively to compensate. After the successful surgery to remove the sacrococcygeal mass, the excised tissue was carefully collected and sent for a detailed histopathological analysis to evaluate the treatment plan for the patient (Figure 4).

Microscopically, the mass exhibited various tissues and inflammatory infiltrates, but no signs of malignancy were observed.

After the operation, the baby was intubated and transferred to the NICU. However, she was successfully extubated on the same day. She received respiratory support through RAM-CPAP (respiratory assist module-continuous positive airway pressure). Her vital signs were normal during her NICU stay, and her surgical wound was regularly dressed. She was transferred from the NICU to the nursery on the third day after the operation.

**Figure 5:**
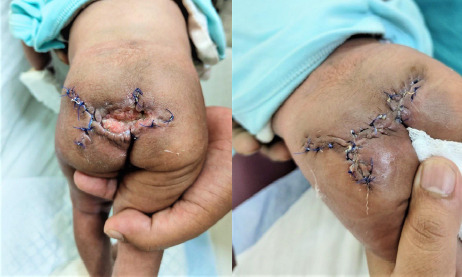
A) Wound dehiscence. B) Secondary closure of the wound.

On the seventh postoperative day, the neonate developed a surgical site wound infection, accompanied by wound gaping. However, there were no neurological deficits, bowel or bladder incontinence in the neonate. Regular dressing was performed, and it was advised to place the baby in the prone and lateral positions to facilitate healing. Despite following the advice, the wound gap persisted. To address this issue, a secondary closure of the wound was performed on the 19th day of life under general anaesthesia (Figure 5).

## DISCUSSION

Sacrococcygeal teratomas, rare extragonadal neoplasms arising in the presacral area, are the most common germ cell malignancy in newborns and young children. Female preponderance has been noted in various literatures with the male:female ratio of 3.4:1. Typically presented as large cysts or solid masses along the body's midline, these tumours consist of tissues derived from two or more primitive germ cells. Although predominantly benign and cystic, sacrococcygeal teratomas have a minimal 1-2% risk of malignant transformation in adulthood.^2^

Altman's classification categorises sacrococcygeal teratomas (SCTs) into four types: type I, an external mass with a small presacral component; type II, an external mass with an intrapelvic component; type III, an external mass with both pelvic and abdominal components; and type IV, an internal mass located within the pelvis and abdomen.^3^ The use of CT (computed tomography) scans and/or MRI (magnetic resonance imaging) is invaluable for diagnosing these lesions and determining their origin, extent, and relationship to the pelvic and abdominal organs.

The neonatal period has been considered the ideal period of surgical resection and not exceeding two months of age. A delay in surgery has been shown to be associated with a higher rate of recurrence or malignancy. Surgical excision is considered curative when the mass is removed along with the coccyx, and confirmation of the teratoma's benign nature is provided by the histopathological report. Pathologically, teratomas are classified as either mature and well-differentiated or immature and poorly differentiated, with the latter having a higher tendency for malignant transformation.^4^ In cases where histopathological examination reveals malignant differentiation, the risk of recurrence can be reduced by administering postoperative chemotherapy and radiotherapy. In our case, the histopathological examination shows a benign tumour, hence no chemotherapy and radiotherapy were advised postoperatively.

Providing anaesthesia to neonates undergoing surgery to remove a sacral teratoma is a challenging task. Several associated abnormalities include hydrocephalus, spina bifida, transposition of great vessels, and cleft lip and palate. Fetal death is often caused by the tumour's arteriovenous shunting, leading to cardiac failure and hydrops, which may require premature delivery.^5^ When hydrops develops, the mortality rate reaches almost 100 percent. In our case, no associated anomalies were detected in the abdominal and pelvic ultrasound and echocardiogram. Specific concerns for these patients include their surgical positioning, blood and fluid loss, and temperature regulation. Maintaining the patient in a prone position is also necessary to prevent wound dehiscence and facilitate wound care in the postoperative period. These patients experience significant blood loss and hypovolemic shock due to the large pelvic venous bed, intra-tumour arteriovenous fistula, and associated coagulopathy. To prevent excessive blood loss, ligating the median sacral vessels early is recommended.

Alternative approaches to managing SCT include techniques like radiofrequency thermal ablation to disrupt the tumour's blood supply and tumour embolization. However, a study has reported unsuccessful attempts to reduce blood flow through the SCT using embolization, balloon occlusion, and sclerosis.^6^ Surgical procedures carry the risk of complications such as damage to the pelvic nerve, rectum, and bladder, which can lead to bowel and bladder dysfunction during later stages of treatment.^7^ There have been reported cases of venous air embolism and cardiac arrest during the surgical procedure.^8^ A caudal block is contraindicated in the presence of a sacrococcygeal teratoma or sacral agenesis. Two instances are documented where the optimal use of ultrasound helped place a sacral intervertebral catheter in two neonates. Long-term follow-up is necessary not only for monitoring tumour recurrence but also for diagnosing and treating potential secondary urinary and/or faecal incontinence.

Wound infection is a commonly observed postoperative complication^9^, and in our case, the baby developed a surgical site wound infection and wound separation, requiring secondary suturing closure at 19 days of life. According to a review, the perioperative death rate was reported as 5.6 percent, with causes of death attributed to haemorrhage, prematurity, birth asphyxia, or tumour rupture. After surgical resection, recurrence rates for both benign and malignant SCTs have been reported to range from 7.5 to 22 percent.^10^ If the coccyx is involved, the likelihood of recurrence is higher. Surgical procedures involving the presacral area have been associated with a higher incidence of lower extremity weakness, paralysis, and complications related to bowel and bladder function.^7^

The management of sacrococcygeal masses can pose challenges in terms of anaesthesia and surgical view, particularly due to associated anomalies and potential blood loss. In our case, the mass was an incidental finding during regular follow-up. The successful management of this case highlights the importance of a multidisciplinary team approach.
